# Sprayed water-based lignin colloidal nanoparticle-cellulose nanofibril hybrid films with UV-blocking ability†

**DOI:** 10.1039/d4na00191e

**Published:** 2024-08-28

**Authors:** Shouzheng Chen, Constantin Harder, Iuliana Ribca, Benedikt Sochor, Elisabeth Erbes, Yusuf Bulut, Luciana Pluntke, Alexander Meinhardt, Bernhard Schummer, Markus Oberthür, Thomas F. Keller, L. Daniel Söderberg, Simone A. Techert, Andreas Stierle, Peter Müller-Buschbaum, Mats K. G. Johansson, Julien Navarro, Stephan V. Roth

**Affiliations:** a Deutsches Elektronen-Synchrotron DESY Notkestraße 85 22607 Hamburg Germany svroth@kth.se stephan.roth@desy.de; b Institute of Wood Science, Universität Hamburg Leuschnerstraße 91 21031 Hamburg Germany; c Forschungs-Neutronenquelle Heinz Maier-Leibnitz FRM II, Technische Universität München Lichtenbergstraße 1 85748 Garching Germany; d Technical University of Munich, TUM School of Natural Sciences, Department of Physics, Chair for Functional Materials James-Franck-Str. 1 85748 Garching Germany; e Wallenberg Wood Science Center (WWSC), Department of Fibre and Polymer Technology, KTH Royal Institute of Technology Teknikringen 56-58 SE-100 44 Stockholm Sweden; f Department of Fibre and Polymer Technology, KTH Royal Institute of Technology Teknikringen 56 SE-100 44 Stockholm Sweden; g Hochschule für Angewandte Wissenschaften (HAW) Hamburg, Department Design Armgartstraße 24 22087 Hamburg Germany; h Centre for X-ray and Nano Science CXNS, Deutsches Elektronen-Synchtrotron DESY Notkestr. 85 22607 Hamburg Germany; i Department of Physics, University of Hamburg Notkestr. 9-11 22607 Hamburg Germany; j Fraunhofer-Institut für Integrierte Schaltungen IIS Flugplatzstr. 75 90768 Fürth Germany; k Institute of X-ray Physics, Goettingen University Friedrich Hund Platz 1 37077 Goettingen Germany

## Abstract

In the context of global climate change, the demand for new functional materials that are sustainable and environmentally friendly is rapidly increasing. Cellulose and lignin are the two most abundant raw materials in nature, and are ideal components for functional materials. The hydrophilic interface and easy film-forming properties of cellulose nanofibrils make them excellent candidates for natural biopolymer templates and network structures. Lignin is a natural UV-shielding material, as it contains a large number of phenolic groups. In this work, we have applied two routes for spray deposition of hybrid films with different laminar structures using surface-charged cellulose nanofibrils and water-based colloidal lignin particles. As the first route, we prepare stacked colloidal lignin particles and cellulose nanofibrils hybrid film through a layer-by-layer deposition. As the second route, we spray-deposite premixed colloidal lignin particles and cellulose nanofibrils dispersion to prepare a mixed hybrid film. We find that cellulose nanofibrils act as a directing agent to dominate the arrangement of the colloidal lignin particles in a mixed system. Additionally, cellulose nanofibrils eliminate the agglomerations and thus increase the visible light transparency while retaining the UV shielding ability. Our research on these colloidal lignin and cellulose nanofibril hybrid films provides a fundamental understanding of using colloidal lignin nanoparticles as functional material on porous cellulose-based materials, for example on fabrics.

## Abstract 2

UV shielding (blue arrow) and visible light transparency (visible light spectrum arrow) of the cellulose nanofibril/lignin colloid thin film are sketched. A SEM image with a sketch of the nanocomposite and hydrated lignin nanoparticle are shown.

## Graphical abstract

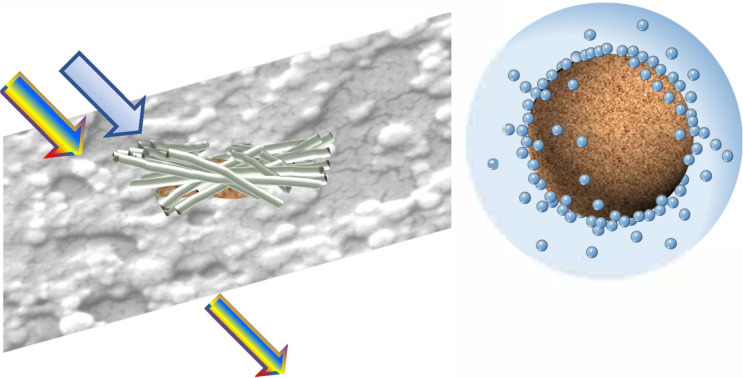

## 1. Introduction

Today, with the challenges of global climate change and pollution from fossil-based products, shifting from a traditional fossil-based materials economy to a sustainable bio-based material economy is feasible and meaningful. Environmentally friendly, sustainable and non-polluting bio-based functional materials will play an important role in the energy and economic transition. Cellulose and lignin, the first and second largest biomaterial reserves in nature, are ideal components for bio-based functional materials, and are also the main constituents of the plant cell walls.^1,2^ Cellulose-based nanomaterials are natural bio-skeletal materials, while lignin-based nanomaterials are natural UV absorbers.^3,4^ Inspired by biomimicry, we can prepare bio-based biomimetic films with a UV shielding function by simulating the structure of plant cell walls with cellulose- and lignin-based nanomaterials.^5^

Nanocelluloses, *e.g.* cellulose nanofibrils (CNFs), cellulose nanocrystals (CNCs), and cellulose nanosheets (CNSs),^6–8^ are considered promising bio-based materials for various applications. Among them, TEMPO (2,2,6,6-tetramethylpiperidine-1-oxyl radical)-mediated oxidized cellulose nanofibrils have received increasing attention and research in recent years due to their high aspect ratio, thermal stability, excellent mechanical properties and good network entanglement.^9–13^ TEMPO-oxidized CNFs with higher surface charge show an increased hydrophilicity, stronger repulsive interactions between fibrils and a better compatibility of the fibrils with other hydrophilic substances (*e.g.* hydrophilic colloids).^14,15^ Interestingly, during the deposition of the CNFs in an aqueous dispersion, the fibrils self-assemble into a dense network structure with the gradual decrease of the water content.^16^ At the same time, the fibrils also entangled with each other to form a dense physical entanglement structure, thereby forming a three-dimensional (3D) porous structure with certain mechanical strength.^17–19^ On the other hand, as reported by Geng *et al.*,^20^ the lower the surface charge of the fibril, the longer was its length. Longer fibrils can achieve a better intertwining network and provide more and uniform attachment sites for colloidal particles, thus improving the homogeneity of the distribution of colloidal particles in the CNF 3D porous structure.^21^ In general, the natural self-assembly properties of aqueous CNF systems lead to excellent 3D porous substrates and framework for flexible functional materials.^22,23^ CNF templates can be functionalized using metal nanoparticles and polymer colloids,^17^ and conductive materials to fabricate bio-based sensors,^19^ and energy conversion devices.^18^

Lignin and its derivatives (*e.g.*, kraft lignin, lignosulfonates) are the second most abundant materials in nature and have great potential for research and application.^24–26^ Lignin is a highly complex and heterogeneous organic macromolecule composed of three phenolic monomers (*para*-coumaryl alcohol, coniferyl alcohol, and sinapyl alcohol units).^27–29^ Due to the large number of phenolic groups, lignin is a natural UV absorber.^30^ Zhang *et al.*^30^ investigated the UV shielding properties of various lignin-based composites and summarized the various factors affecting the UV shielding effect and chromaticity of lignin. The factors affecting the UV shielding performance are the phenolic group content of lignin, the shape and size of lignin particles, and the polydispersity index (PDI) of lignin particles. Among them, the phenolic group content of lignin is directly proportional to the UV shielding performance, *i.e.* the higher the phenolic group content, the stronger the UV shielding performance. The PDI is inversely proportional to the color brightness, *i.e.*, the lower the PDI value, the lighter the color of lignin. Spherical and smaller-sized lignin particles are beneficial to improve UV shielding ability and also to obtain lighter colors of lignin. Kraft lignin has more phenolic content than other types of lignin, making it the ideal raw material for UV shielding.^30^ However, its complex and disordered macromolecular structure and water-insoluble properties limit its application. Common methods used to dissolve lignin include sulfonation, succinylation and aqueous lignin colloidal particles. The latter approach provides a better strategy for the use of kraft lignin as a UV additive due to the spherical shape, low polydispersity index and water-soluble properties of the particles.^31,32^ In the process of preparing colloidal lignin particles, the more commonly used method is the solvent-shifting method. This method involves replacing the organic solvent of an organic solution of kraft lignin with water.^33,34^ Österberg *et al.*^35^ reported a method for the preparation of aqueous kraft lignin solutions by the solvent-shifting method, using organic solvents such as acetone or tetrahydrofuran. This process resulted in the self-assembly of kraft lignin molecules into stable spherical nanoparticles with hydrophilic groups on the surface, with a diameter of 50–100 nm.^36–38^ This strategy solves the problem of the water insolubility of kraft lignin and the heterogeneity of kraft lignin macromolecules, thereby greatly broadening the practical applications of kraft lignin.

Spray deposition is a commonly used fabrication method in industry due to its advantages of being fast, low-cost and suitable for large-scale fabrication.^39,40^ We choose to use TEMPO-oxidized CNF and colloidal lignin particles (CLP) as raw materials to prepare hybrid films by spray deposition, which allows to obtain the CNF and CLP hybrid films with different laminar structure and nanostructure by designing the layer-by-layer spraying parameter. Liu *et al.* found that different nanostructures of lignin colloidal particles (such as size and periodic arrangement) correspond to different optical properties.^33^ The effect of different nanostructures of CLP in hybrid films on their optical properties is significant. However, there are few reports on different nanostructures in CLP films and CLP-based composite films obtained by spray-deposition. Thus, we focused on characterizing the nanostructures induced by spray-deposition and different particle interactions in CLP-based films and constructing their correlation with wettability as well as optical properties.

In this work, water-soluble cellulose nanofibrils (CNFs) are prepared by TEMPO oxidation. Water-soluble colloidal lignin particles (CLPs) with a particle size of around 40 nm are prepared by solvent shifting method, aiming at solving the problems of macromolecular heterogeneity and water-insolubility of kraft lignin. We prepare four types of thin films: two pure CLP films with different densities and two hybrid films of CLP and CNF with different structures by spray deposition. Scanning electron microscopy (SEM), atomic force microscopy (AFM) and grazing incidence small angle X-ray scattering (GISAXS) are used to characterize the nanostructures and morphology of the thin films induced by the self-assembly process of CNF and CLP during spray deposition. The dynamic wettability and UV-Vis transmittance of the films are investigated by measuring the dynamic contact angle and UV-Vis spectrum, respectively, as well as the effect of the obtained nanostructures on the UV-Vis transmittance.

## 2. Experimental section

### 2.1 Preparation of aqueous cellulose nanofibrils (CNF) solution

The wood pulp fibers were oxidized with 2,2,6,6-tetramethylpiperidin-1-yloxy (TEMPO), to provide fibrils with a negative surface charge by oxidizing the –CH_2_OH groups to COOH.^9^ We followed the same procedure as in the previous research report.^20^

In this work, CNF with a negative surface charge of 400 μmol g^−1^ with a radius of around 2 nm and a length of around 600 nm was chosen. CNFs were prepared from chemically bleached wood fibers (a mixture of 60% Norwegian spruce and 40% red pine, provided by Domsjo AB, Sweden). For the preparation of CNFs with negative surface charge densities of 400 μmol g^−1^, cellulose pulp fibers (1 g) were suspended in a 0.05 mol L^−1^ sodium phosphate (Sigma-Aldrich) buffer (90 mL, pH 6.8), containing TEMPO (16 mg, 0.1 mmol) and sodium chlorite (Sigma-Aldrich) (80%, 1.13 g, 10 mmol). A 2 mol L^−1^ sodium hypochlorite solution (0.5 mL) after dilution to 0.1 mol L^−1^ with the 0.05 mol L^−1^ sodium phosphate buffer was added to the suspension. The suspensions were subsequently stirred at 500 rpm for 1 h. The TEMPO-oxidized pulp fibers were washed thoroughly with deionized water by filtration. After the chemical pretreatment, aqueous suspensions of the fibers were passed through a high-pressure homogenizer. At the end of homogenization, CNF suspensions with a concentration larger than 5 g L^−1^ were obtained. In an additional step, the unfibrillated and agglomerated fiber bundles were removed from the CNF suspensions. The gel-like suspensions were diluted to 0.07 wt% by adding deionized water, then treated by mechanical mixing (12 000 rpm, 10 min, Ultra Turrax, IKA, Germany) and ultrasonication (10 min, BANDELINSONOPULS HD/UW 2070, Germany). Finally, the solution was centrifuged (5000 rpm, 60 min, Rotina 420, Hettich GmbH & Co. KG, Germany), and the supernatant was taken to obtain the CNF dispersion.^20^

### 2.2 Synthesis of colloidal lignin particle (CLP)

The kraft lignin used in this work was softwood LignoBoost kraft lignin from StoraEnso. First, 3 g of kraft lignin was dissolved in 300 mL of a mixture of acetone and water (volume ratio acetone to water 3 : 1). Then, the suspension was filtered by filter paper (Whatman GF/F, pore size 0.7 μm) to remove the insoluble parts and impurities. The kraft lignin/acetone/water solution was rapidly added to 600 mL of deionized water during heating (*T* = 70 °C) and stirring (700 rpm, Magnetic Stirrers RET control-visc, IKA, Germany) for 4 h to evaporate all the acetone in the solution. The heating temperature was controlled at around 70 °C to reach the saturated vapor pressure of acetone. Water was added to a final volume of 825 mL, thus yielding an aqueous CLP suspension of about 0.75 wt%.^36^

**Fig. 1:**
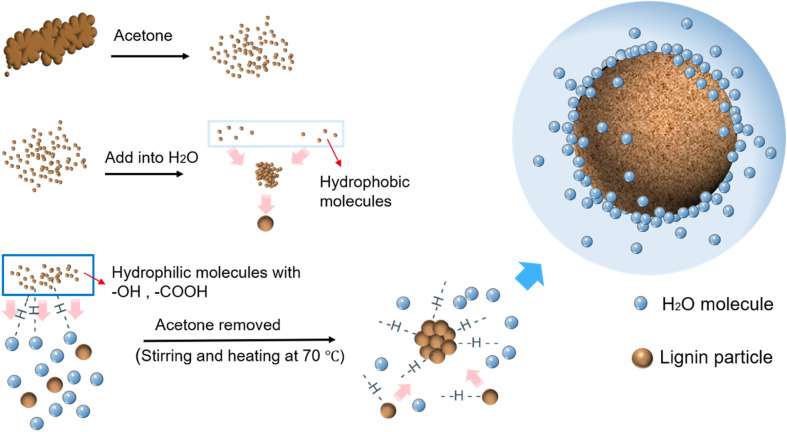
Schematic diagram of the self-assembly mechanism of water-based spherical lignin colloidal nanoparticles (CLP) prepared by solvent shifting method. In this process, water is added as a strong polar solvent and acetone is gradually evaporated. The kraft lignin self-assembles into nanoparticles.

The formation of the CLP is as follows (Fig. 1). Firstly, when added to water, the aqueous environment will cause the hydrophobic lignin molecules to aggregate, forming primary lignin aggregates. With the gradual reduction of the acetone content, the outermost layer of the primary lignin aggregates is wrapped by hydrophilic lignin molecules containing a large number of hydroxyl and carboxyl groups to form spherical lignin colloidal particles.^32^ When acetone was completely removed, an aqueous kraft lignin colloidal solution was obtained. Hydrogen bonds can be created between the hydrophilic groups on the surface of the colloidal lignin particles and water molecules. The colloidal lignin nanoparticles repel each other due to their negative surface charges.^35^

### 2.3 Spray deposition

The substrates for spray deposition were silicon (Si) wafers (one-side polished, boron-doped, resistivity 1–30 Ω.cm, orientation (100), Si-Mat, Germany) of size 2 cm × 2 cm and ultraviolet (UV) transparent quartz glass wafers (PGO, Germany) of 2.5 cm × 2.5 cm. Samples deposited on the glass wafers were used for UV-Vis measurement. The cleaning procedure was the same for both as follows: ultrasonic cleaning with isopropyl alcohol (98%) for 15 min, followed by cleaning with ethanol (96%, all Sigma-Aldrich, Germany) and ultrapure water (18.2 MΩ cm, Milli-Q, Merck KGaA, Germany), respectively. The wafers were pretreated by UV-ozone cleaner (Ossila, UK) for 15 min, which aims to increase the free energy of the surface and to reduce the surface tension of the dispersion on the surface of the wafer to facilitate spray deposition. The substrate temperature is set at 120 °C for spraying.

**Fig. 2:**
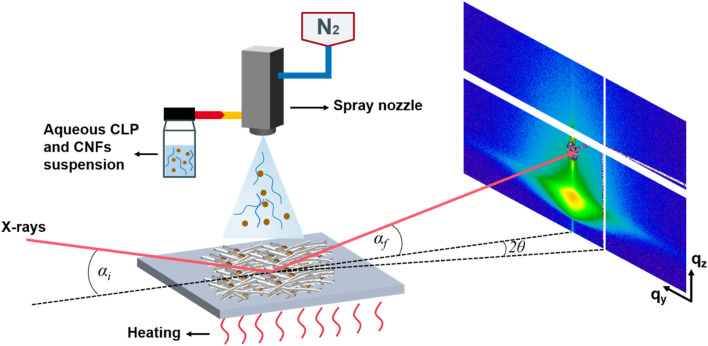
Sketch of the spray deposition and the grazing incidence small-angle X-ray scattering (GISAXS) geometry. *α*_i_ is the incident angle, *α*_f_ is the exit angle, 2*θ* denotes the out-of-plane scattering angle, *q*_y_, *q*_z_ denote the wavevector transfers in horizontal and vertical direction.

Spray deposition (Fig. 2) was performed by laboratory spray equipment (Compact JAUD555000, Spray Systems, Germany). The solution was filled into a glass container and connected to the spraying equipment *via* a siphon. N_2_ pressure for spraying was 1.5 bar. A heating plate was attached to the base plate and the temperature can be set. The distance between the nozzle and substrate (NSD) was set as 20 cm for all depositions.

### 2.4 Layer formation and designation

In this article, there are four different film layer formations: CLP, CLP/H_2_O, CLP on CNF and CLP/CNF. For CLP and CNF hybrid films, we use two routes to design the CLP and CNF stacks. In the first route, we use a layer-by-layer (LBL) spray deposition alternating CNF and CLP to prepare CNF and CLP hybrid films with CNF as the flexible, lowermost layer for CLP. For the second route, a premixed aqueous suspension of CNF/CLP is deposited to prepare CNF/CLP mixed film. The following designation is used for the sprayed samples.

#### 2.4.1 CNF 20P

Samples obtained using a 0.07 wt% CNF dispersion pulsed 20 times on silicon wafer.

#### 2.4.2 CLP 20P

Samples obtained using a 0.75 wt% CLP dispersion pulsed 20 times on the silicon wafer.

#### 2.4.3 CLP/H_2_O 20P

Samples was obtained by diluting the original CLP dispersion 20 times with water (to about 0.037 wt%) and pulsing it 20 times on a silicon wafer.

#### 2.4.4 CLP/CNF 20P

Samples obtained by pre-mixing 0.037 wt% CLP/H_2_O dispersion and 0.07 wt% CNF dispersion in a 1 : 1 ratio and spraying 20 pulses on silicon wafer.

#### 2.4.5 CLP ON CNF 20P

A sample of the 0.037 wt% CLP/H_2_O dispersion pulsed 20 times on a CNF film (the CNF film is obtained by pulsing the 0.07 wt% CNF dispersion 20 times on a silicon wafer).

### 2.5 Characterization

#### 2.5.1 Scanning electron microscopy

The SEM used for the experiments was a JEOL JSM-7900F. The procedure of sample measurement was covering the samples in the sputtering chamber with 5 nm gold and ensuring the electric connection with carbon clay.^41,42^ The samples were transferred in the vacuum chamber and measured with different magnifications. All the samples were measured under 9.634 × 10^−5^ Pa.

#### 2.5.2 Atomic force microscopy

Topographical images were obtained *via* atomic force microscopy (AFM) using a RTESPA-150 and cantilever type Dimension Icon AFM (Bruker) with NanoScope V controller.^43^ Image dimensions are as follows: Scan size: 2 × 2 micrometers. Scan speed: 0.5 Hz. Resolution: 1024 × 1024 pixels. For the measurement of surface roughness, three different regions were selected for measurement and the mean value as well as the error was calculated. The area of each measured region is 500 × 500 nm. For thickness measurements, the film was scratched and the scratches were imaged by atomic force microscope. The resulting steps were linearly integrated and leveled to obtain the thickness. The data analysis was performed by Gwyddion.^44^

#### 2.5.3 Grazing incidence small-angle X-ray scattering

The grazing incidence small-angle X-ray scattering (GISAXS) experiments were performed at the MiNaXS/P03 beamline of the PETRA III storage ring at DESY, Hamburg,^45^ during two different experimental campaigns (first campaign CLP 20P and CLP/H_2_O 20P; second campaign CLP ON CNF 20P and CLP/CNF 20P). A sketch of the GISAXS geometry is shown in Fig. 2. The sample-to-detector distance was 6195 ± 1 mm/3603 ± 1 mm, respectively. The energy of the X-rays was 11.9 keV (*λ* = 1.044 Å)/11.8 keV (*λ* = 1.041 Å), respectively. The incident angle *α*_i_ was set at 0.32°/0.4° for GISAXS. 2D GISAXS data were collected in all cases by a PILATUS 2M detector (Dectris Ltd., Switzerland) with a pixel size of 172 × 172 μm^2^. Two separated, point-like beamstops were used to shield the direct and the specular reflected beam.

#### 2.5.4 Contact angle measurements

Dynamic contact angle measurements were performed using the OCA35 (Data physics, Germany) contact angle measuring system. Measurements were taken at three different locations for each sample to calculate the average mean as well as the error for analysis. The volume of each water droplet is 3 μL. The dynamic process of the contact angle was recorded for 240 s, and the contact angle was measured and recorded at 20 s intervals. The measurement of the contact angle is performed by the SCA20 (Data physics, Germany) software.

The dynamic variation of the contact angle is fitted by the following equation:^46^1
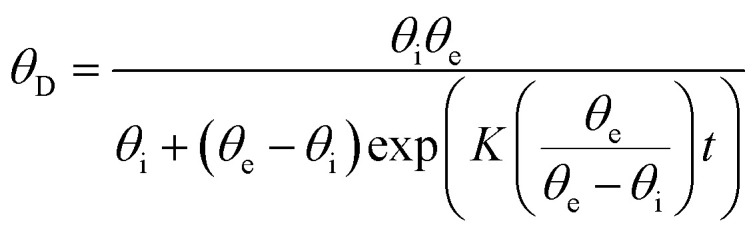
with *θ*_i_ as the initial CA at *t* = 0 s, *θ*_e_ as CA when equilibrium is reached (in this article at *t* = 240 s), *K* as the spreading and penetration rate constant, and *t* as the absorption time.

#### 2.5.5 Ultraviolet-visible transmittance

Ultraviolet-visible (UV-Vis) transmittance measurements were performed using a Varian Cary 5E UV-Vis-NIR Spectrophotometer (Varian, Germany). The UV-Vis data of each sample were selected from three different positions of the same sample, and the average of the three measurements was calculated.

The measured absorbance data of the films are converted to obtain the transmittance using the Beer–Lambert equation:

2 *A* = lg(1/*T*)

*A* is the absorbance, *T* is the transmittance ratio (transmittance).

## 3. Results and discussion

### 3.1 Surface morphology

**Fig. 3:**
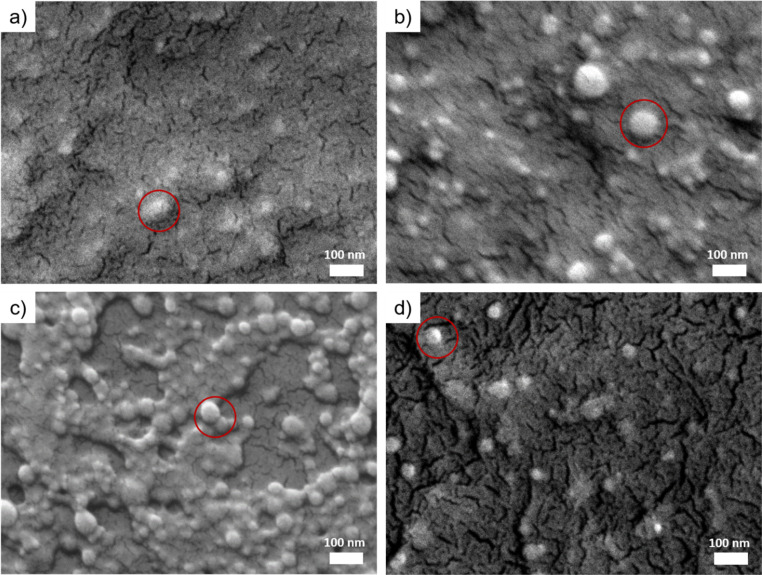
SEM images of CLP-based samples. (a) CLP 20P. (b) CLP/H_2_O 20P. (c) CLP on CNF 20P. (d) CLP/CNF 20P. The red rings indicate colloidal lignin nanoparticles.

SEM is used to characterize the surface morphology and the distribution of the CLP particles in all films obtained by spray-deposition. As shown in Fig. 3a, the morphology of individual colloidal lignin nanoparticles is hardly observed in the CLP 20P film due to the high concentration of colloidal lignin particles in the dispersion, resulting in the aggregation of the deposition particles to form a dense laminar structure. In the CLP/H_2_O 20P film (Fig. 3b), a large number of spherical CLP particles with diameters of 30–50 nm can be seen. The degree of agglomeration of CLP particles is smaller than that of the CLP 20P samples (Fig. 3a). This observation indicates that the agglomeration of CLP particles is effectively reduced by diluting the CLP dispersion, and highly dispersed CLP can be deposited. For the Fig. 3c shows that, the CLP particles deposited on the CNF substrate are uniformly distributed and less agglomerated. In the CLP ON CNF 20P film (Fig. 3d), the CLP particles are not agglomerated, and the distance of lignin nanoparticles is larger compared to CLP 20P, CLP/H_2_O 20P, and CLP on CNF 20P.

**Fig. 4:**
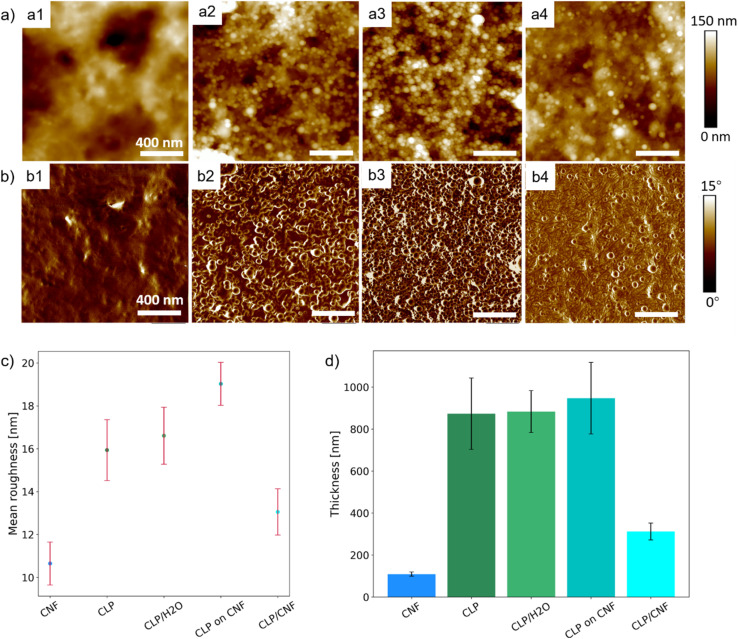
(a) AFM height images of CLP-based samples: a1, a2, a3, a4 are CLP 20P, CLP/H_2_O 20P, CLP on CNF 20P, CLP/CNF 20P, respectively. (b) AFM phase images of CLP-based samples: b1, b2, b3, b4 are CLP 20P, CLP/H_2_O 20P, CLP on CNF 20P, CLP/CNF 20P, respectively. (c) Mean roughness distribution of CNF and CLP-based samples. The mean roughness of CNF 20P, CLP 20P, CLP/H_2_O 20P, CLP on CNF 20P, CLP/CNF 20P are 11 ± 1 nm, 16 ± 1 nm,17 ± 1 nm, 19 ± 1 nm, 13 ± 1 nm, respectively. (d) Thickness distribution of CNF and CLP-based samples. The thickness of CNF 20P, CLP 20P, CLP/H_2_O 20P, CLP on CNF 20P, CLP/CNF 20P are 109 ± 10 nm, 873 ± 170 nm, 883 ± 100 nm, 947 ± 170 nm, 312 ± 40 nm, respectively.

To better understand the surface properties and the three-dimensional (3D) configuration and the growth process induced by the spray deposition of different dispersions, the surface roughness and thickness of the samples were characterized by AFM. Fig. 4a1 and b1 shows that the surface of the CLP 20P sample is flat and no CLP particles are observed. In contrast, individual and inter-clustered CLP particles are present on the surface of the CLP/H_2_O 20P sample. At the same time, these inter-clustered CLP particles create an inhomogeneous porous structure (Fig. 4a2 and b2). This difference in film structure and surface flatness is caused by the concentration of the initial dispersion (around 0.75 wt%). CLP 20P has a lower surface roughness than CLP/H_2_O 20P (see Fig. 4c). From Fig. 4a3 and b3, it can be seen that there are numerous aggregated spherical CLP particles on the surface of CLP ON CNF 20P, which has a similar surface morphology as the CLP/H_2_O 20P sample. However, the surface roughness of the CLP ON CNF 20P sample is significantly higher than that of CLP 20P and CLP/H_2_O 20P (see Fig. 4c). This finding is attributed to the initial roughness of the CNF substrate translating to inhomogeneity of the CLP particles during the deposition and dispersion process; due to the size of the colloidal lignin particles, they cannot penetrate into the voids of the underlying CNF layer as is the case for smaller colloids.^21^ Among the CLP-based samples, the CLP/CNF 20P sample has the lowest surface roughness, which is attributed to the fact that CNF dominates the homogeneous dispersion of CLP particles in the 3D porous structure of CNF during the deposition process (Fig. 4a4 and b4). CNF also acted as a “mediator” to reduce the agglomeration between CLP particles, while CNF and CLP particles contributed to the surface properties together to reduce the surface roughness.

From Fig. 4d, the thickness of the CLP 20P sample is similar to that of the CLP/H_2_O 20P sample, which indicates that the growth process of the CLP deposited layer depends on the number of pulses: more pulses give an increased thickness of the CLP particles layer. The concentration of the initial CLP dispersion determines the degree of densification and porosity of the deposited layer: the higher the concentration of the initial CLP dispersion, the denser the CLP particle layer. The thickness of the CLP ON CNF 20P sample is the highest among the four samples, which is approximately equal to the sum of the thickness of the CLP/H_2_O 20P and the CNF 20P samples. The thickness of the CLP/CNF 20P sample is significantly lower than that of the other CLP-based samples because the CNFs dominate the self-assembly process of the CLP particles during the deposition process. Random and irregular voids caused by inhomogeneous accumulation of CLP agglomerate are eliminated. The structure of other CLP-based samples is characterized by the aggregation and stacking of CLP particles, whereas the structure of CLP/CNF 20P is more homogeneous, resulting in a significantly lower film thickness.

### 3.2 Inner film morphology

**Fig. 5:**
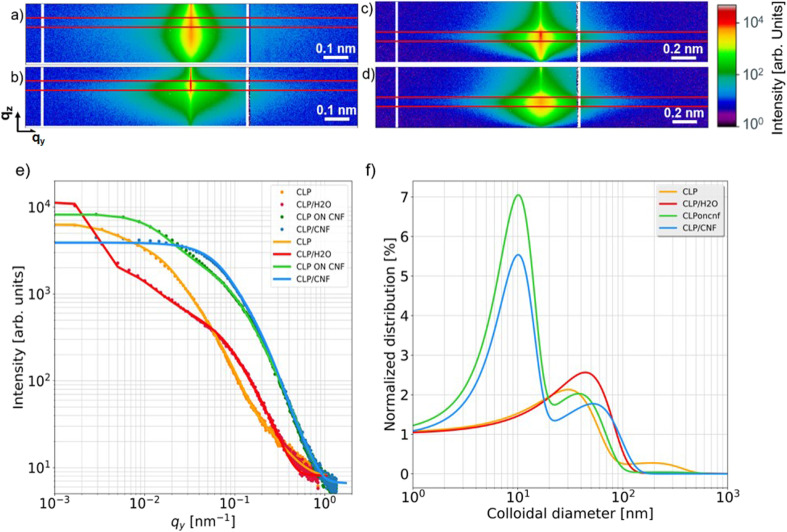
2D GISAXS data with region of the horizontal line cuts in the Yoneda region of (a) CLP 20P, (b) CLP/H_2_O 20P, (c) CLP ON CNF 20P, (d) CLP/CNF 20P (e) horizontal line cut data (symbols) and corresponding fits (solid lines). (f) Normalized distribution of colloidal diameters as extracted from the model fits.

Grazing incidence small-angle X-ray scattering (GISAXS) is a powerful tool for characterizing surface and subsurface nanostructures of thin films.^47–50^ GISAXS allows to obtain the surface and interfacial structure of the thin films, such as the shape, dimension, and polydispersity of the nanostructures.^51,52^ To relate the SEM and AFM findings to the inner film nanostructures, all films are measured using GISAXS and analyzed to extract the different nanostructures in the films.^16^ GISAXS pattern are shown in Fig. 5a–d and fits to the cuts in Fig. 5e. CLP 20P shows three nanostructures with diameters of 30 ± 10 nm, 190 ± 3 nm and 850 ± 23 nm, respectively (for details of the fits see the ESI, Fig. S1†. CLP/H_2_O 20P has two CLP nanostructures with diameters of 44 ± 3 nm and 360 ± 9 nm, respectively. The corresponding diameters and the distribution of the colloidal lignin nanoparticles are presented in Fig. 5f. Both CLP 20P and CLP/H_2_O have a main contribution of lignin colloids with diameters around 40 nm. This is inferred from the contribution of medium-sized (diameter is from 190 nm to 360 nm) lignin agglomerates. The presence of medium-sized agglomerates is inevitable due to the dense deposition of lignin particles. The formation of medium-sized structures is also related to the roughness of the film. The lower roughness of CLP 20P has a smaller medium-sized structure (diameter 190 ± 16 nm). We can see from Fig. 5f that only CLP 20P has a higher distribution at the larger structure (diameter 850 ± 60 nm), which may be caused by even larger agglomerates of colloidal lignin nanoparticles caused by high concentrations or by lignin particles fused together after reaching their glass transition temperature (*T*_g_).^53^Fig. 4a1 also indicates such larger agglomerations with sizes well beyond 500 nm, corroborating the large scale found by modelling the GISAXS data. CLP ON CNF 20P shows three nanostructures with diameters of 38 ± 25 nm, 10 ± 55 nm and 240 ± 9 nm, respectively. CLP/CNF 20P has two structures with diameters of 52 ± 1 nm and 10 ± 3 nm, respectively. Following Fig. 5f, the first structure of both samples is related to individual colloid lignin particle with a diameter of about 50 nm. The second structure in both samples is a colloid with a diameter of about 10 nm, consistent with CNF agglomerates,^16^ where the CNF structure in CLP on CNF 20P has a large error of 55 nm in diameter, which is equal to the diameter of a single CLP particle. This error value is supposed to be caused by the special structural composite formed when lignin particles are initially deposited on the porous structure of the CNF, *i.e.* with CNF wrapped around an individual CLP. CLP/CNF 20P has only two structures of lignin particles and CNF. The reason is that, according to Fig. 4a4 and b4, the agglomeration is greatly reduced due to the increase of the distance between CLP particles due to the uniform distribution of CLP particles in the porous structure of CNF established during the deposition and drying of the mixed dispersion.

### 3.3 Wetting and imbibition behavior

**Fig. 6:**
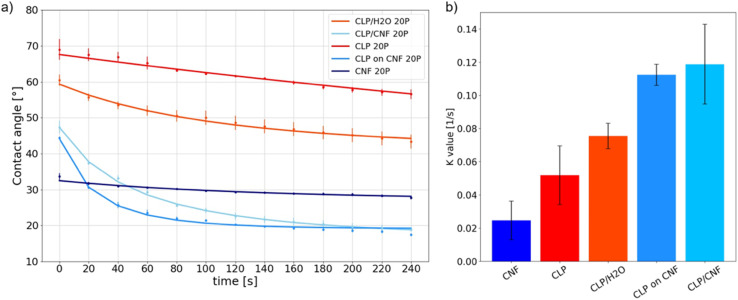
(a) Dynamic contact angle of CLP 20P, CLP/H_2_O 20P, CLP on CNF 20P, CLP/CNF 20P and CNF 20P with 20 pulses. (b) *K* value determined from the analysis of the dynamic contact angle data.

First of all, the contact angle values at *t* = 0 s (directly after deposition) of CNF 20P is 34 ± 1° while that of CLP 20P is 69 ± 5°, see Fig. 6a. Both CNF and CLP films have hydrophilic interfaces, and CNF has a higher hydrophilicity. The temporal evolution of the dynamic contact angle of CNF 20P is nearly linear with the smallest *K* value, see Fig. 6b. The attachment and diffusion of water droplets on the surface are stable, possibly related to its low surface roughness (Fig. 4c). However, the attachment and diffusion process of water droplets on the surface of thin films are affected by various factors. They include the hydrophilicity of the material, the surface roughness, the pore structure, and the surface nanostructure.^54^ The hydrophilicity and the porous structure favor imbibition of water.

The dynamic curve of CLP 20P tends to be linear while that of CLP/H_2_O 20P is nonlinear. The *K* value of CLP/H_2_O 20P is higher than that of CLP 20P. As shown in Fig. 4c and d, CLP 20P has lower roughness and denser structure than CLP/H_2_O 20P. The higher roughness leads to an unstable attachment of droplets, which affects the diffusion process. CLP/H_2_O 20P has a looser structure, *i.e.* more inter-particle voids. A higher number of void structures with hydrophilic interfaces will accelerate the penetration process of droplets and thus accelerate diffusion. We can infer that the difference in dynamic processes between CLP 20P and CLP/H_2_O 20P films is affected by the combination of their surface roughness and void structure.

For CLP on CNF 20P, the static contact angle at 0 s is 44 ± 1°. However, between 0 s and 20 s, the contact angle is in a sudden downward trend. Combined with Fig. 4c, the higher surface roughness of CLP on CNF 20P leads to instability in the initial attachment process, which may cause droplet slippage at the initial stage and thus lead to a sudden drop. The underlying CNF layer has a more hydrophilic interface, which exacerbates the permeation effect of the film and thus the instability of droplet spreading. The surface of CLP/CNF 20P is dominated by CNF and CLP together. Hence the contact angle at 0 s is intermediate between the values of the contact angles of the CNF and CLP films. CLP/CNF 20P has a low surface roughness (Fig. 4c), and at the same time CLP/CNF 20P has a hydrophilic interface dominated by CNF and a uniform pore structure. We thus hypothesize that the hydrophilic pore structure dominates the dynamic wetting process of CLP/CNF 20P leading to fast droplet diffusion.

### 3.4 Correlation to optical properties

**Fig. 7:**
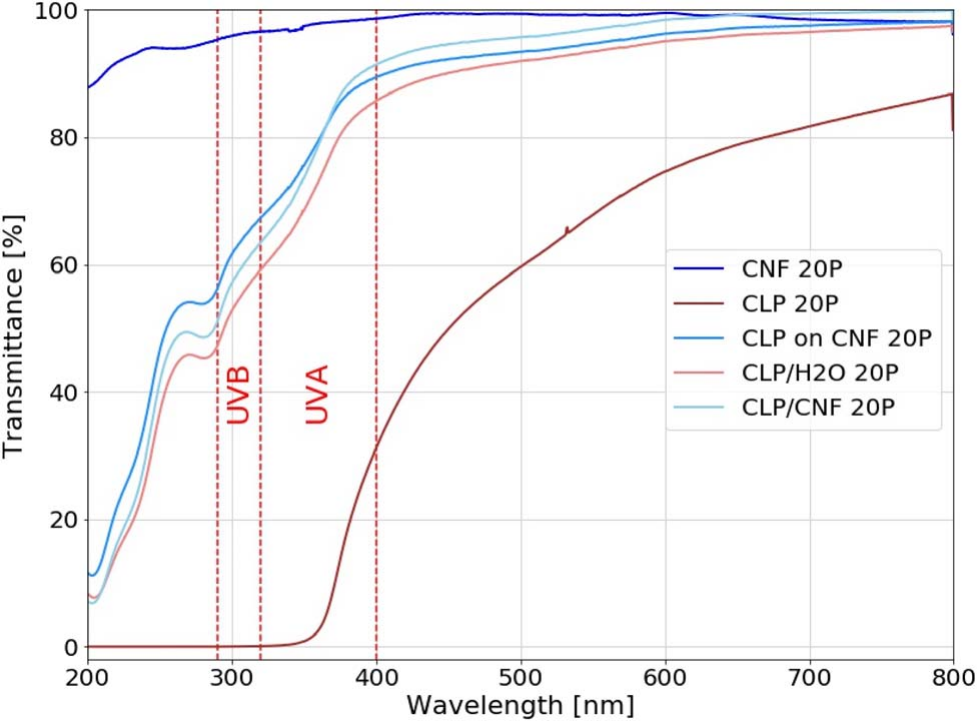
UV-Vis transmittance of CNF 20P, CLP 20P, CLP/H_2_O 20P, CLP ON CNF 20P and CLP/CNF 20P samples.

The overall UV shielding capacity of a film as well as its transparency to visible light is related to its nanostructure. The UV shielding capacity is positively correlated with the density of CLP particles per unit, whereas the transparency to visible light is negatively correlated with the dimension and polydispersity of the nanostructure. By designing specific nanostructures, the UV-Vis properties of the films can be optimized. The transparency to visible light should be high while maintaining the UV shielding ability. In order to analyze the effect of the different nanostructures on the UV-Vis transparency of the CLP and CNF hybrid films, we used CLP/H_2_O 20P, CLP on CNF 20P and CLP/CNF 20P for comparative analyses, while CNF 20P and CLP 20P were used as reference samples, see Fig. 7.

The CNF 20P sample has poor UV shielding ability but is almost transparent to visible light. The CLP 20P sample shielded completely the UV light between *λ* = 280–370 nm, which covers the UVB region (280–315 nm) and partially the UVA region (315–400 nm). However, the UV transmittance gradually increases above 370 nm, which is due to the UV absorbance properties of CLP. This is an inherent property of CLP particles (ESI†). The transmittance of CLP 20P samples to visible light increases gradually with increasing wavelength, with an average transmittance of around 70%.

Regarding the transmittance *T*_UV_ in the UV radiation region (*λ*_vis_ = 280–400 nm), the following order can be established: *T*_UV_(CLP ON CNF) > *T*_UV_(CLP/CNF) > *T*_UV_(CLP/H_2_O), while the transmittance in the visible light region is *T*_Vis_(CLP/CNF) > *T*_Vis_(CLP ON CNF) > *T*_Vis_(CLP/H_2_O). We found that CLP ON CNF and CLP/H_2_O have similar CLP morphology (Fig. 4a2, b2 and a3, b3), but CLP ON CNF has lower UV shielding capabilities. This is probably due to the roughness of the CNF substrate that leads to the inhomogeneity of the CLP particle deposition layer. This results locally in a low amount of CLP particles within the UV penetration pathway. Meanwhile, the transparency of CLP ON CNF to visible light (400–800 nm) was slightly better than that of CLP/H_2_O. CLP/CNF has better UV shielding ability compared to CLP ON CNF 20P, and the transparency to visible light is significantly higher than that of the other films. As shown in Fig. 4a4 and b4, the CLP particles in CLP/CNF are not agglomerated and well-distributed. The visible light transparency was greatly improved while maintaining the UV shielding ability. CLP/CNF 20P has lower thickness than other CLP-based films, so the UV shielding capacity per unit thickness of CLP/CNF 20P is higher than that of CLP/H_2_O 20P sample.

## 4. Conclusion

By investigating the nanostructure and morphology of CLP nanoparticles and sprayed films, we find that agglomeration effects exist between CLP particles, but also that the dilution of the dispersion with water can eliminate this agglomeration. For the pure CLP deposited layer, the concentration of the CLP dispersion determines the density of the CLP layer obtained by spray deposition because of the small and uniform CLP size. The number of spray pulses determines the thickness of the CLP layer. For flexible CNF substrates with a certain roughness, the initial deposition phase leads to uneven deposition and dispersion of CLP particles, which increases the surface roughness of the hybrid film. The mixed system of CNF and CLP particles forms a heterogeneous structure dominated by CNFs' entanglement network, which allows the CLP particles to be uniformly dispersed in the pore structure of the CNF. It is significantly more homogenous than the other systems studied. During the spray deposition process, CNF acts as a directing agent to distribute CLP particles uniformly in the 3D porous structure of the CNF film. It eliminates the aggregation of the CLP particles and no CLP agglomerates are found in CLP/CNF mixed system by GISAXS characterization.

To correlate structure and function, we analyze the dynamic wetting process of the films by surface roughness and porous structure. Higher surface roughness leads to an instability of the droplet attachment and thus affects the diffusion process, while the porous structure with hydrophilic interfaces accelerates droplet penetration and thus accelerates the droplet diffusion process. With the help of UV-Vis transmittance, we find that the nanostructures of CNF and CLP mixing systems reduces the aggregation density of CLP particles and increases the visible light transparency while retaining the UV shielding ability. The research related to CLP particles and CLP and CNF hybrid films of this article is applicable to a wide range of substrates requiring UV protection and provides a reference for the wider study and application of CLP and CNF hybrid films, which may act as model systems to further integration in apparel and wearables.

## Conflicts of interest

There are no conflicts to declare.

## Supplementary Material

NA-006-D4NA00191E-s001

## Data Availability

The data that support the findings of this study are available on request from the corresponding author, SVR, upon reasonable request. Additionally, the data supporting this article have been included as part of the ESI.†
