# Supplementary material for: Sprayed water-based lignin colloidal nanoparticle-cellulose nanofibril hybrid films with UV-blocking ability
Source: Nanoscale Adv. 2024 Aug 28;6(20):5031–41. doi: 10.1039/d4na00191e (PMC11376076; doi:10.1039/d4na00191e)
Supplement: NA-006-D4NA00191E-s001 [file NA-006-D4NA00191E-s001.pdf]

Supplementary Information (SI) for Nanoscale Advances.
This journal is © The Royal Society of Chemistry 2024

Supplementary Information for

Sprayed Water-Based Lignin Colloidal Nanoparticle-Cellulose Nanofibril Hybrid Films with UV-Blocking Ability

Shouzheng Chen[1,2,3], Constantin Harder[1,4], Iuliana Ribca[5,6], Benedikt Sochor[1], Elisabeth Erbes[1,11], Yusuf Bulut[1,4], Luciana Pluntke[1,7], Alexander Meinhardt[8,9], Bernhard Schummer[10], Markus Oberthuer[7], Thomas F. Keller[8,9], L. Daniel Söderberg[5,6], Simone Techert[1,11], Andreas Stierle[8,9], Peter Müller-Buschbaum[4], Mats K. G. Johansson[5,6], Julien Navarro[2] and Stephan V. Roth[1,5,6*]

1. Deutsches Elektronen-Synchrotron DESY, Notkestraße 85, 22607 Hamburg, Germany

2. Institute of Wood Science, Universität Hamburg, Leuschnerstraße 91, 21031 Hamburg, Germany

3. Forschungs-Neutronenquelle Heinz Maier-Leibnitz FRM II, Technische Universität München, Lichtenbergstraße 1, 85748 Garching, Germany

4. Technical University of Munich, TUM School of Natural Sciences, Department of Physics, Chair for Functional Materials, James-Franck-Str. 1, 85748 Garching, Germany

5. Wallenberg Wood Science Center (WWSC), Department of Fibre and Polymer Technology, KTH Royal Institute of Technology, Teknikringen 56-58, SE-100 44 Stockholm, Sweden

6. Department of Fibre and Polymer Technology, KTH Royal Institute of Technology, Teknikringen 56, SE-100 44 Stockholm, Sweden

7. Hochschule für Angewandte Wissenschaften (HAW) Hamburg, Department Design, Armgartstraße 24, 22087 Hamburg, Germany

8. Centre for X-ray and Nano Science CXNS, Deutsches Elektronen-Synchtrotron DESY, Notkestr. 85, 22607 Hamburg, Germany

9. Department of Physics, University of Hamburg, Notkestr. 9-11, 22607 Hamburg, Germany

10. Fraunhofer-Institut für Integrierte Schaltungen IIS, Flugplatzstr. 75, 90768 Fürth, Germany

11. Institute of X-ray Physics, Goettingen University, Friedrich Hund Platz 1, 37077 Goettingen, Germany

Corresponding author:

*svroth@kth.se; stephan.roth@desy.de

GISAXS parameters

a) Set-up of CLP 20P and CLP/H_2O 20P:

The sample-to-detector distance (SDD) was kept at 6195 mm in GISAXS. The energy of X-ray was 11.9 keV (λ=1.044 Å) with a beam center X = 848 [pixel], Y = 222 [pixel]. One beam stop was used to shield the specular reflected beam. The incident angle α_i was set at 0.32° for GISAXS. 2D GISAXS data were collected by a PILATUS 2M detector (Dectris Ltd., Switzerland) with a pixel size of 172 × 172 μm^2.

b) Set-up of CLP on CNF 20P and CLP/CNF 20P:

The sample-to-detector distance (SDD) was kept at 3602.99 mm in GISAXS. The energy of X-ray was 11.8 keV (λ=1.041 Å) with a beam center X 880 [pixel], Y 301 [pixel]. One beam stop was used to shield the specular reflected beam. The incident angle α_i was set at 0.4° for GISAXS. 2D GISAXS data were collected by a PILATUS 2M detector (Dectris Ltd., Switzerland) with a pixel size of 172 × 172 μm^2.

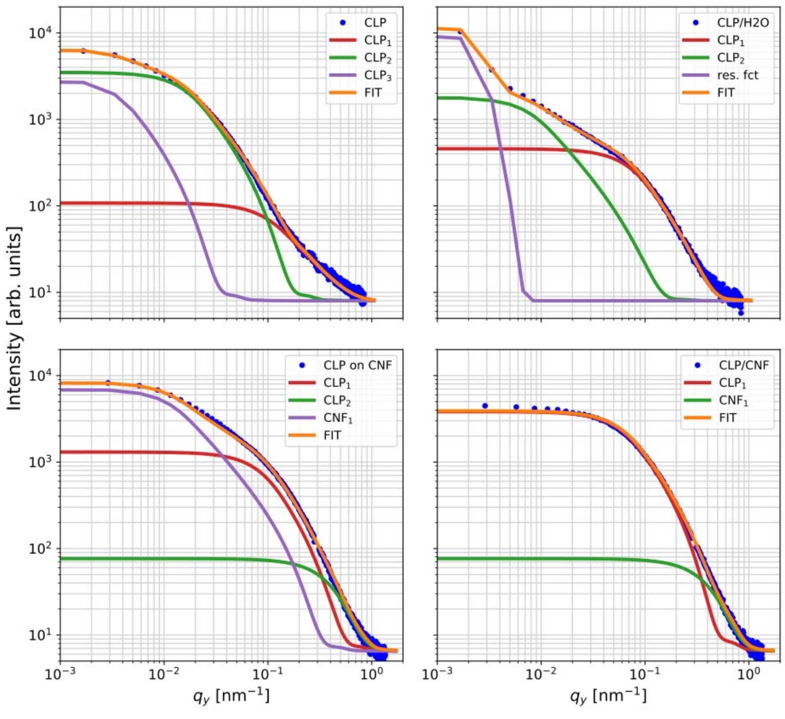

Fig. S1 Fitted horizontal line cuts data from the 2D GISAXS data. CLP 20P sample have three lignin nanostructure CLP_1, CLP_2, CLP_3 detected, which have a different diameter and distribution. CLP/H2O 20P sample have two lignin nanostructures. CLP on CNF 20P sample have the similar two lignin nanostructure with CLP/H2O samples detected, but also one CNF structure CNF_1. CLP/CNF 20P sample have only one lignin nanostructure and one CNF nanostructure, because of the homogeneity of the nanoparticles and their distribution. The resolution function was only detected in CLP/H2O 20P sample as res. fct, that due to the very high roughness of lignin samples and the resolution function was mixed into nanostructure signal in other three samples.

Table S1. Diameter and polydispersity of different structures in the CLP-based samples from Fig. 1.

	CLP 20P	CLP/H2O 20P	CLP on CNF 20P	CLP/CNF 20P
Diameter [nm] 1st	30 ± 10	44 ± 3	38 ± 25	52 ± 1
Polydispersity 1st	0.8 ±0.3	0.7 ± 0.1	0.8 ± 0.9	0.7 ± 0.1
Diameter [nm] 2nd	190 ± 16	360 ± 9	10 ± 55	10 ± 3
Polydispersity 2nd	0.8 ±0.1	0.9 ± 0.1	0.4 ± 5.0	0.4 ± 0.8
Diameter [nm] 3rd	850 ± 60	None	240 ± 9	None
Polydispersity 3rd	0.8 ± 0.1	None	0.9 ± 0.1	None

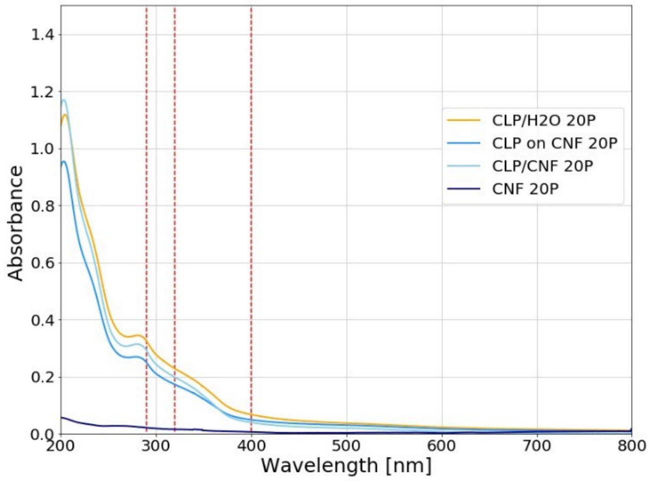

Fig. S2 UV-Vis Absorbance of CNF 20P, CLP/H_2O 20P, CLP on CNF 20P and CLP/CNF 20P samples.
